# Patterns of Vertebrate Diversity and Protection in Brazil

**DOI:** 10.1371/journal.pone.0145064

**Published:** 2015-12-17

**Authors:** Clinton N. Jenkins, Maria Alice S. Alves, Alexandre Uezu, Mariana M. Vale

**Affiliations:** 1 Instituto de Pesquisas Ecológicas, Nazaré Paulista, São Paulo, Brazil; 2 Departamento de Ecologia, Universidade do Estado do Rio de Janeiro, Rio de Janeiro, Rio de Janeiro, Brazil; 3 Departamento de Ecologia, Universidade Federal do Rio de Janeiro, Rio de Janeiro, Rio de Janeiro, Brazil; Macquarie University, AUSTRALIA

## Abstract

Most conservation decisions take place at national or finer spatial scales. Providing useful information at such decision-making scales is essential for guiding the practice of conservation. Brazil is one of the world’s megadiverse countries, and consequently decisions about conservation in the country have a disproportionate impact on the survival of global biodiversity. For three groups of terrestrial vertebrates (birds, mammals, and amphibians), we examined geographic patterns of diversity and protection in Brazil, including that of endemic, small-ranged, and threatened species. To understand potential limitations of the data, we also explored how spatial bias in collection localities may influence the perceived patterns of diversity. The highest overall species richness is in the Amazon and Atlantic Forests, while the Atlantic Forest dominates in terms of country endemics and small-ranged species. Globally threatened species do not present a consistent pattern. Patterns for birds were similar to overall species richness, with higher concentrations of threatened species in the Atlantic Forest, while mammals show a more generalized pattern across the country and a high concentration in the Amazon. Few amphibians are listed as threatened, mostly in the Atlantic Forest. Data deficient mammals occur across the country, concentrating in the Amazon and southeast Atlantic Forest, and there are no data deficient birds in Brazil. In contrast, nearly a third of amphibians are data deficient, widespread across the country, but with a high concentration in the far southeast. Spatial biases in species locality data, however, possibly influence the perceived patterns of biodiversity. Regions with low sampling density need more biological studies, as do the many data deficient species. All biomes except the Amazon have less than 3% of their area under full protection. Reassuringly though, rates of protection do correlate with higher biodiversity, including higher levels of threatened and small-ranged species. Our results indicate a need for expanded formal protection in Brazil, especially in the Atlantic forest, and with an emphasis on fully protected areas.

## Introduction

Knowledge of where species occur, in particular the species more vulnerable to extinction, is essential for planning conservation and research efforts. Several recent studies have focused on global scale mapping of biodiversity and priorities for conservation [1–3]. Such efforts can be useful, particularly in guiding international conservation attention. Much of conservation planning, however, takes place not at the global scale but rather at national or regional scales. This may not always be ideal, since the countries with more financial resources for conservation are often not the countries with the most biodiversity. As well, nationally designed conservation strategies may not be as efficient as a globally ideal strategy [4]. Nevertheless, most conservation takes place within a nation centered reality.

We explored the specific case of Brazil, one of the most biodiverse countries in the world. For three vertebrate groups (birds, mammals, and amphibians), we examined patterns of richness, degree of threat, and endemism within the country. To understand the potential limitations of the existing data, we then explored how spatial bias in collection localities may influence the perceived patterns of diversity. Under the assumption that areas of high biodiversity should receive more conservation attention, we conclude with a comparison of how well protected areas overlap with places deemed important for biodiversity.

Analyses of biodiversity at a national level often focus on a single taxonomic group, such as birds. They are also typically at coarse scales (e.g., biomes in Marini & Garcia 2005)[5]. Explicit spatial analyses of biodiversity have been rare in Brazil at a national level, but there are examples at a regional level for birds [6–8], mammals [9], primates [10–12], amphibians [13–16], squamates [17], and vertebrates in general [18,19]. Sobral et al. (2014)[20] recently did a national level analysis, although limited to select predatory birds and mammals. With the recent and rapid increases in spatial biodiversity data, now is an opportune time to examine what we know about Brazil’s biodiversity patterns, where the gaps might be, and whether such knowledge is informing conservation.

Choosing an appropriate measure of biodiversity is crucial, for not all species are of equal concern. Wide-ranging species drive patterns of overall species richness [21]. Such species are, however, typically not of conservation concern and many may even benefit from degraded habitats [22,23]. Consequently, using overall species richness to guide conservation could be misleading. A more useful guide are smaller-ranged species or local endemics, which tend to be more vulnerable and in need of conservation attention [24]. We focused most of our analyses on these species.

Our multi-taxa approach also enabled us to identify similarities and differences between taxa and between biodiversity metrics, identifying areas where multiple groups present a higher biodiversity importance. This allowed an assessment of whether the biodiversity conservation efforts adopted, in this case protected areas, correlate with high biodiversity areas or if there are gaps that need more conservation actions.

## Methods

Polygon range data for birds are from BirdLife International and NatureServe [25], and for mammals and amphibians from the International Union for the Conservation of Nature [26]. When the original range data included subspecies, we merged them into a single species map. Threatened species were those considered vulnerable, endangered, or critically endangered in the IUCN Red List [27]. We did not separately evaluate species listed as threatened on the national Red List maintained by ICMBio [28]. There were inconsistencies in the taxonomies used, and we preferred to focus on globally threatened species. We defined endemic species as those having at least 90% of their range within Brazil and no part of their range extending more than 50km beyond the border. Small-ranged species are those with ranges smaller than the median for that taxon in Brazil (2,250,813 km^2^ for birds, 1,230,901 km^2^ for mammals, 66,979 km^2^ for amphibians). We did not include marine mammals or seabirds, as our focus is terrestrial species. We did not include species considered Extinct or Extinct in the Wild. Species diversity maps were rendered at a spatial resolution of 10×10 km and use an equal area projection (South America Albers Equal Area Conic). We considered a species whose range overlapped any part of a grid cell to be present for that cell. Analyses used ArcGIS 10.2 (ESRI).

To explore how spatial bias in collections may influence the perceived patterns of diversity, we used species occurrence records from the Global Biodiversity Information Facility (GBIF) (www.gbif.org) and the national *species*Link dataset (splink.cria.org.br). We downloaded all records in Brazil that were georeferenced and listed as having no known coordinate issues. To account for minor spatial uncertainties near the border, we extracted the GBIF data using a 10km buffer beyond the Brazilian border. Thus, ours is a liberal estimate of the amount of locality data within the country, possibly including some points from neighboring countries.

To illustrate sampling effort, we created a network of Thiessen polygons for each taxon and database (GBIF and *species*Link). In the network, each locality point generates one polygon and everything within the polygon is closer to its locality point than to any other record in the dataset. Consequently, the larger the polygon, the lower the collection density because the entire area is represented by a single locality. Polygon size, therefore, can be interpreted as a surrogate for sampling effort. To evaluate whether there was congruence in polygon sizes between GBIF and *species*Link we ran a correlation analysis using the corrected number of degrees of freedom from Dutilleul’s method for spatial autocorrelation adjustment using SAM 4.0 [29].

For each of the six biomes (Amazon, Cerrado, Atlantic Forest, Caatinga, Pantanal, and Pampa) and 27 states in Brazil, we calculated the percentage of area occupied by protected areas. Biome and state boundaries are from the Brazilian Geography and Statistics Institute [30].

For protected areas, we considered three categories with different degrees of protection, two based on conservation of natural resources (fully protected areas and sustainable use areas) and one based on indigenous culture preservation (indigenous lands). Fully protected areas (Proteção Integral, in Portuguese) are completely restrictive of human use except for research and educational visits, while sustainable use areas allow for some types of extractive use. Indigenous lands are created to guarantee the right of indigenous people to their land, and often have few restrictions related to biodiversity. Protected areas data are from the Brazilian Ministry of Environment (www.mma.gov.br).

Because the boundaries of fully protected areas and areas of sustainable use sometimes overlap, we prioritize the most restrictive type. Therefore, in any overlap between the two we classified the overlapping area as fully protected. Indigenous land comprises two types, indigenous reserves and indigenous territories. For our purposes, we combined them into one class. From the originally downloaded database, we removed indigenous land that were not yet implemented and those in study or with no definitive boundaries. Indigenous lands also overlap with other types of protected areas and when necessary we calculated their coverage separately.

To compare protection rates with patterns of biodiversity, we calculated four metrics of biodiversity based on species categories (all species, Brazil endemics, small-ranged, threatened). Each of these metrics is a summary layer across the three taxa analyzed. For each category, we first rescaled the richness values for each individual taxa (e.g., birds, mammals, amphibians) to a scale of 0 (minimum richness) to 1 (maximum richness). We then summed the rescaled layers of the three taxa for the relevant category, resulting in a potential value from 0 to 3 for any given biodiversity metric. To compare biodiversity with protection, we binned the pixels by intervals of 0.2 and then calculated the proportion of the pixels with those values that were within protected areas.

## Results

**Fig 1:**
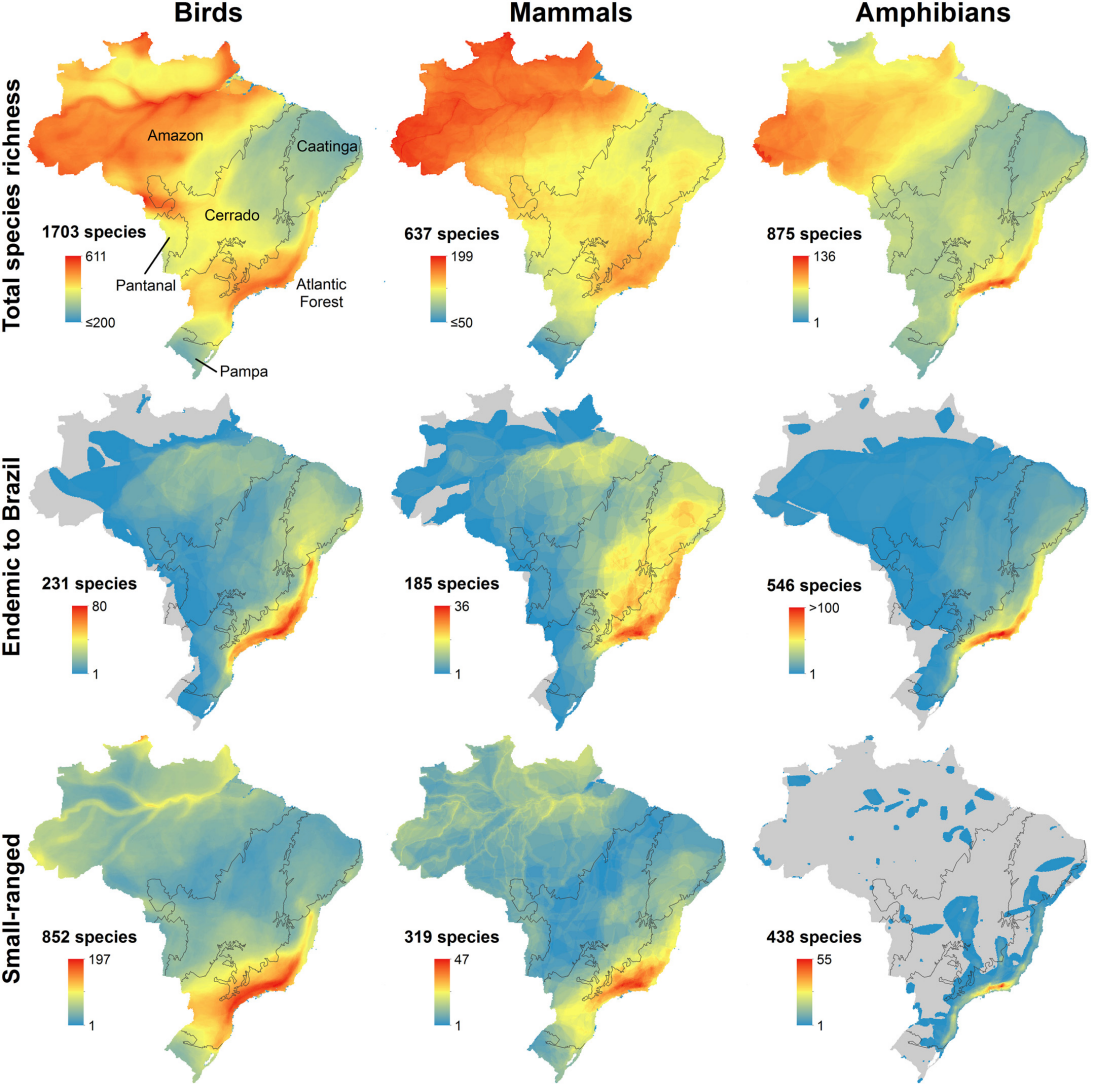
Patterns of diversity for birds, mammals, and amphibians in Brazil. Gray lines delimit biomes.

In terms of overall species richness, the Amazon and Atlantic Forest clearly have the highest values (Fig 1). This is consistent across all three taxa. Amphibians do have a more pronounced difference between the forested Amazon and Atlantic Forest biomes and the drier Cerrado and Caatinga biomes, which show substantially lower richness. For species endemic to Brazil, the Atlantic Forest biome dominates, especially the region from southern Bahia to São Paulo (Fig 1). The Pampa and Pantanal biomes have few Brazil endemics.

Patterns for small-ranged species, defined as those with ranges smaller than the median for that taxon in Brazil, are similar to those for endemics (Fig 1). This is not surprising since the two groupings share many species. It is overwhelmingly the case for amphibians, where 401 of the 438 small-ranged amphibians are also endemic to the country. Nevertheless, patterns differ in important ways. For amphibians, the peak diversity appears in the state of Rio de Janeiro and the contiguous region of São Paulo state, an even more focused concentration than that seen with endemics. For birds and mammals, the trend is the opposite, with a slightly more generalized pattern for small-ranged species than seen with endemics, but with the peak diversity still in the Atlantic Forest, particularly in its southern portion.

**Fig 2:**
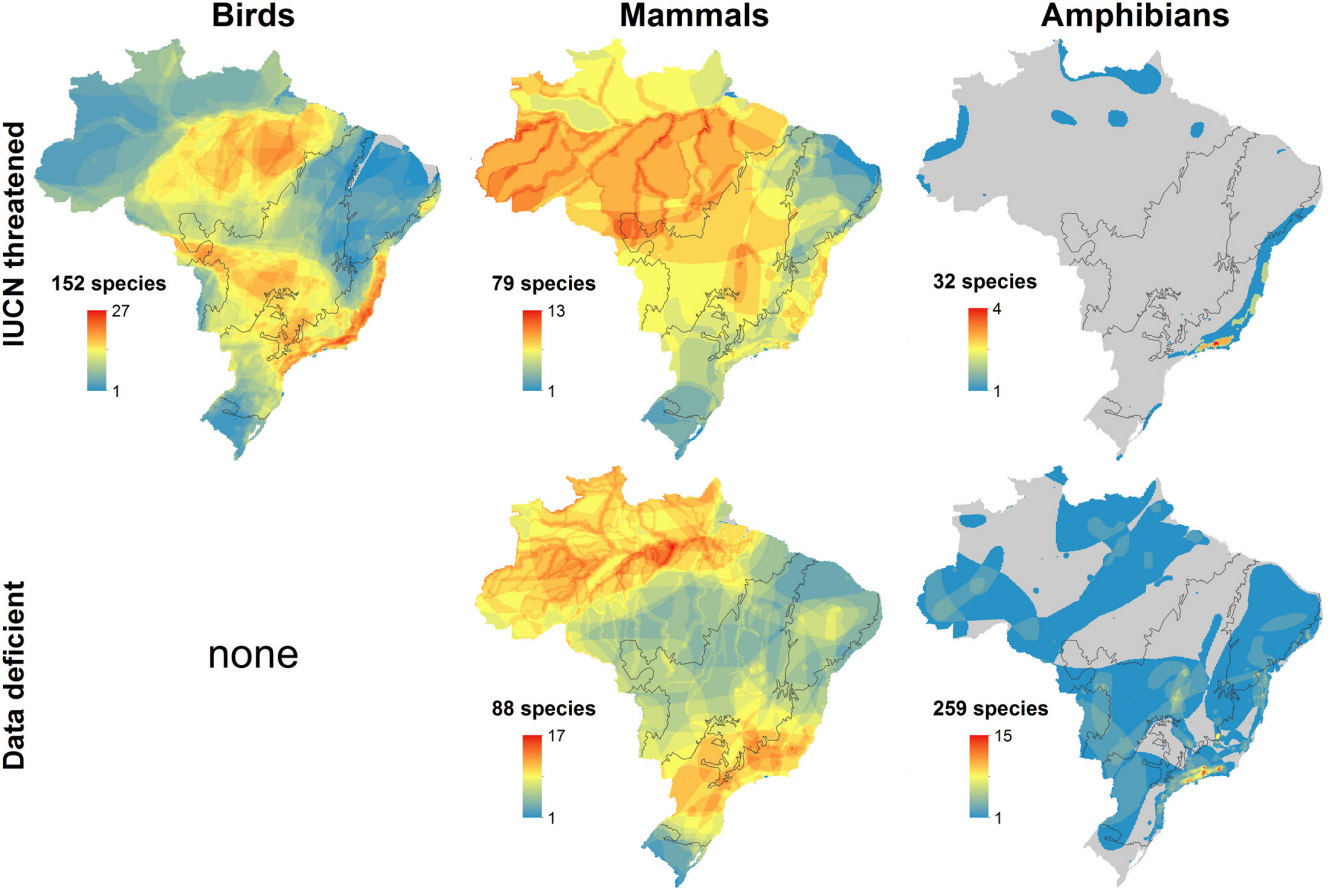
Patterns of diversity for threatened and data deficient species in Brazil. Threatened species are those listed as Vulnerable, Endangered, or Critically Endangered on the IUCN Red List.

Patterns for IUCN threatened species do not show a consistent pattern across taxa (Fig 2). For birds, the geographic pattern is similar to overall species richness (Fig 1), with higher concentrations of threatened species in the Atlantic Forest but also high numbers in parts of the Amazon and Cerrado. Mammals show a more generalized pattern across the country, but with a higher concentration in the Amazon, particularly along rivers. For amphibians, few species are formally listed as threatened, with most of those in the Atlantic Forest. Data deficient mammals occur across the country, concentrating in the Amazon and the southeast Atlantic Forest, and there are no data deficient bird species in Brazil (Fig 2). There are, however, a very large number of data deficient amphibians (259 species, 30% of the total). These are widespread across the country, but with an especially high concentration in the far southeast (Fig 2).

**Fig 3:**
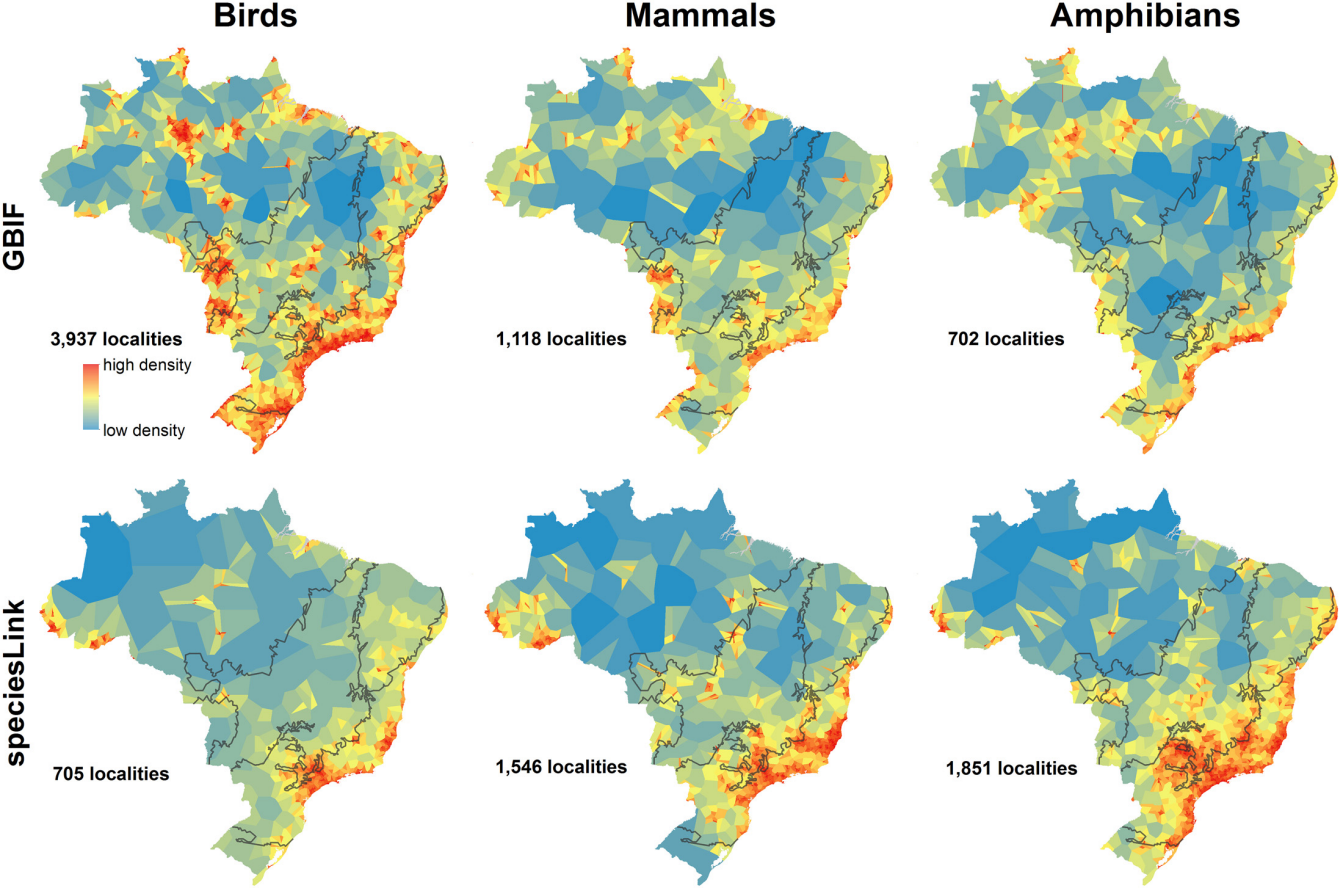
Thiessen polygon networks to represent sampling density. Maps derive from data from the Global Biodiversity Information Facility (GBIF) and from Brazil’s *species*Link site. Polygon colors correspond to their size and use a Jenks natural breaks grouping for the color stretch. Gray lines delimit biomes.

The comparison between the GBIF and *species*Link databases showed that the distribution of locality data for the taxa under study is concentrated in relatively few places (Fig 3 and S1 Fig). Many of the areas with high concentrations of locality data are near major cities in the more developed southeast (e.g., Rio de Janeiro, São Paulo, Vitória) and along major rivers in the Amazon, leaving vast poorly sampled areas in the north and central part of the country (Fig 3).

That there is spatial sampling bias is not new [31]. More interestingly, the GBIF and *species*Link databases are inconsistent with one another. GBIF has far more localities for birds (3,937 in GBIF vs. 705 in *species*Link, Fig 3), while *species*Link has far more for amphibians (702 in GBIF vs. 1,851 in *species*Link, Fig 3). The two databases also do not always highlight the same areas as having the most data. For example, some areas with high concentrations of bird records in GBIF have few or no records for birds in *species*Link (Fig 3). The reverse is true for amphibians. There are significant correlations between the two databases for all taxa (Spearman’s r = 0.2 to 0.34; p<0.05 for all comparisons), but they are relatively low considering that the two databases have similar goals.

**Fig 4:**
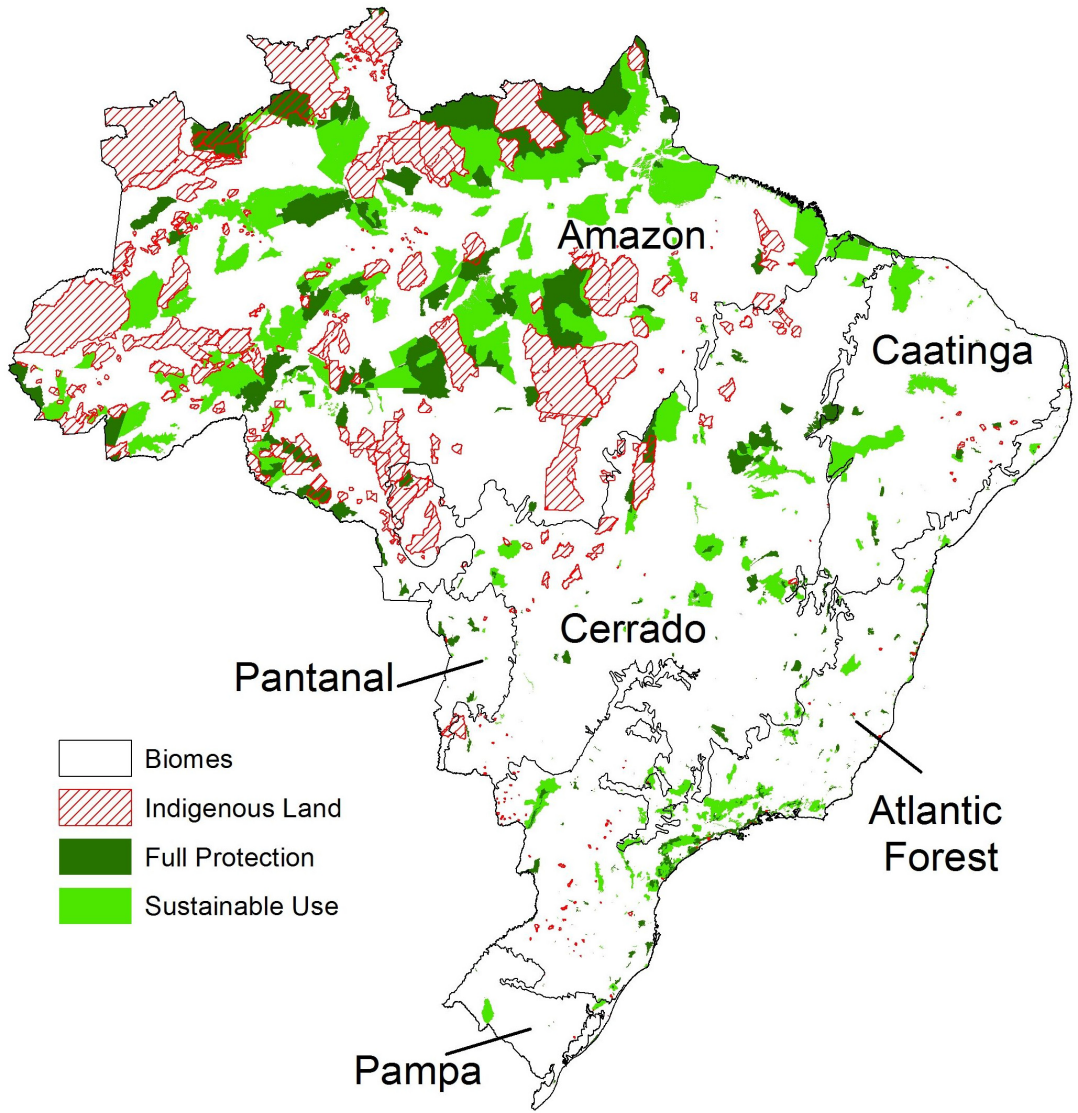
Distribution of protected areas and indigenous lands in Brazil. Some indigenous lands overlap other protected area categories, and some sustainable use areas overlap fully protected areas.

Recognizing that our knowledge of biodiversity patterns does have limits, has what we do know informed conservation decisions? If so, one might expect that protected areas would coincide with places of higher value for preserving species. In Brazil, 28.3% of the land area is under some type of formal protection, with 6% under full protection, 10.8% in sustainable use, and 12.2% in indigenous lands (Fig 4, Table 1, see S1 Table for protection by state). Considering all categories, the Amazon has the highest percentage of its area occupied by protected areas (47.7%). The other five biomes have much lower percentages, with the Pampa being the most neglected at just 2.9% protected (Table 1). All biomes except the Amazon have substantially less protected than the internationally agreed upon goal of 17% protection (Aichi Target 11) [32]. If we consider only the Fully Protected areas, the situation is drastically worse. All of the biomes outside the Amazon have less than 3% of their area under Full Protection (Table 1).

**Table 1:** Percentage of area protected in each biome. Table order is by declining biome size. Because indigenous lands sometimes overlap other protected areas, and some protected areas overlap others, the total percentage protected (FP+SU+IL) is not a simple sum of its components.

Biome	Area (km^2^)	FP (%)	SU (%)	FP+SU (%)	IL (%)	FP+SU+IL (%)
Amazon	4,198,943	9.9	16.5	26.4	22.5	47.7
Cerrado	2,040,060	2.7	4.9	7.6	4.1	11.4
Atlantic Forest	1,117,857	2.4	5.8	8.2	0.3	8.4
Caatinga	827,956	1.1	6.3	7.5	0.2	7.7
Pampa	165,810	0.4	2.5	2.9	0.0	2.9
Pantanal	151,179	2.9	0.3	3.2	1.4	4.6
Brazil	8,501,804	6.0	10.8	16.8	12.2	28.3

FP—Full Protection,

SU—Sustainable Use,

IL—Indigenous Land.

**Fig 5:**
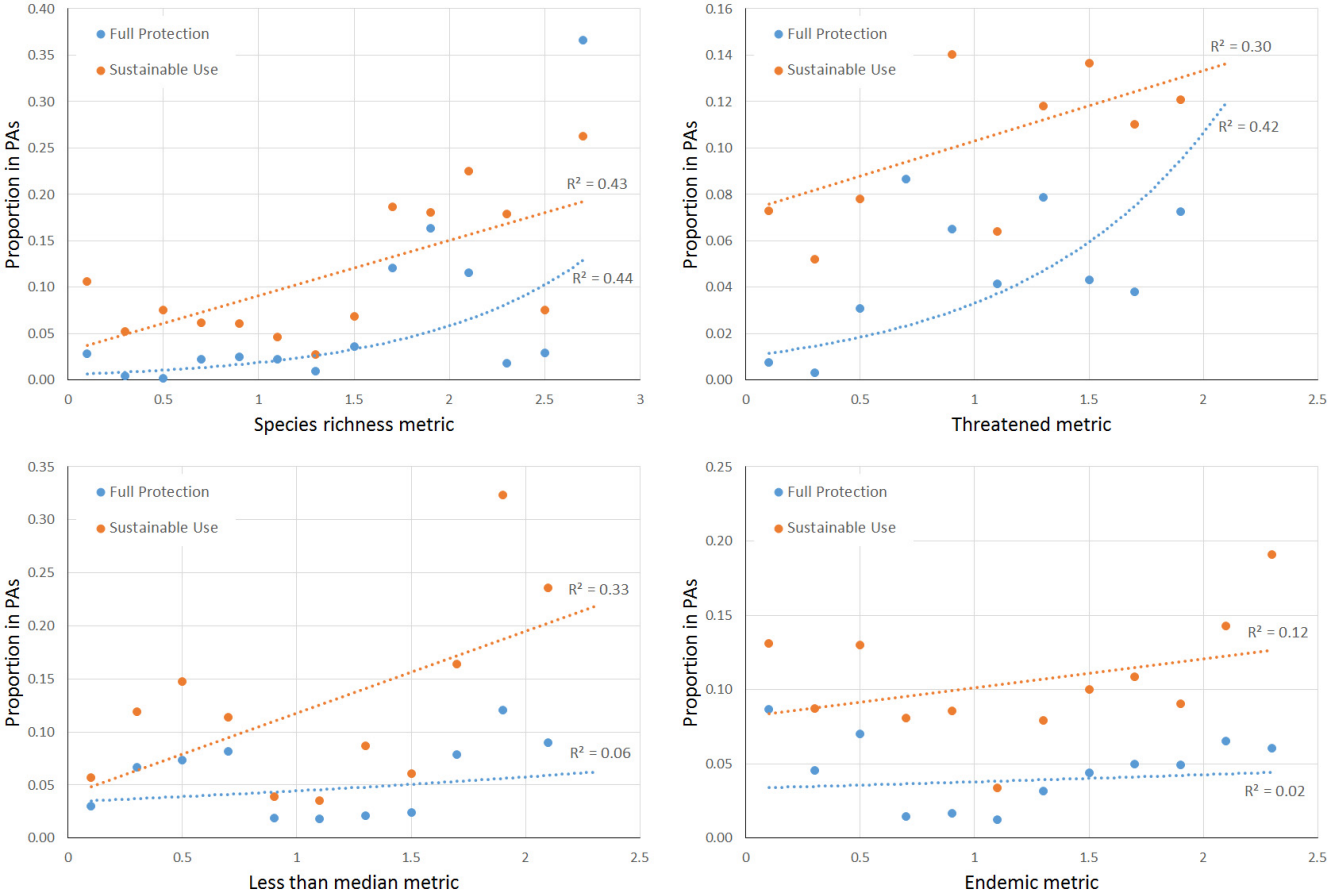
Rates of protection versus multi-taxa metrics of biodiversity. Data points correspond to intervals of 0.2 on the potential 0 to 3 scale of each biodiversity metric. For example, a point at 0.5 represents all of the areas in Brazil that had a value between 0.4 and 0.6 for that particular metric. Lines represent regressions. PAs—Protected Areas.

Protected areas show a positive relationship with all four summary indices of biodiversity (Fig 5). Total species richness shows the strongest link to percent coverage of protected areas, mainly due to relatively high protected area coverage in the species rich Amazon (Fig 5). The more informative indices based on small-ranged, endemic, and threatened species also show positive relationships with protected area coverage, although lower in general, and the endemism index has a very low R^2^ for both types of protected areas (Fig 5). In all cases, the proportional coverage by fully protected areas is substantially lower than that by sustainable use areas. We did not evaluate indigenous areas because their goal is not to protect biodiversity, so we have no *a priori* reason to expect them to follow biodiversity patterns.

## Discussion

Due to its megadiverse nature, much of the Brazilian territory is of high importance for global conservation. Nevertheless, all countries face choices on which specific places to protect. In Brazil, the species likely to be of more conservation concern, the endemics and other small-ranged species, strongly concentrate in the Atlantic Forest. The Atlantic Forest is isolated from other forested biomes and is well-known for hosting many endemic species [33,34] and for being a high-priority biodiversity hotspot [35]. Other biomes have potential explanations for their relatively low concentrations of Brazil endemics and small-ranged species. The Amazon extends far beyond the Brazilian border, and so many currently known Amazonian species, which tend to have large ranges in general, reach into neighboring countries [36]. Because it is relatively understudied, the Amazon also likely still has many smaller-ranged and endemic species awaiting discovery. The Cerrado savanna is a biodiversity hotspot and a well-known center of plant endemism, although less so for terrestrial vertebrates [35,37]. The Cerrado is almost all within Brazil but with small portions in Bolivia and Paraguay, which may partially explain its relatively low richness of endemic vertebrates. The Caatinga, a semi-arid region with xeric vegetation in northeastern Brazil, is the only biome entirely inside Brazil. It has lower total diversity, but seemingly should have relatively high endemism of species adapted to its extreme conditions. The Caatinga, however, has traditionally been considered to be poor in endemics (e.g., Andrade-Lima 1982)[38]. Nevertheless, the region is poorly inventoried and studies are starting to reveal a concealed endemism [39,40].

The pattern of endemics and other small-ranged species concentrating in the Atlantic Forest contrasts somewhat with that of documented threatened species, which show a broader geographic dispersal. There are potential explanations. In the Atlantic Forest and Cerrado, habitat loss and fragmentation tend to be the major causes of threat and are activities that affect nearly all species. In the Amazon, however, some wide-ranging species likely have sufficient habitat but face threats from other sources, such as hunting, disease, or invasive species. This may particularly be the case among birds and mammals in Brazil (e.g., Peres 2000)[41].

Of concern are the many data deficient mammals and amphibians, a substantial number of which are probably threatened. Of the 875 mapped amphibians in the country, only 32 (<4%) are listed as globally threatened, mostly in the Atlantic Forest. That is far lower than the current global rate for amphibians of 31% threatened. However, 259 (30%) of Brazil’s amphibians are listed as data deficient (Fig 2). Were they better known, many of these species would likely qualify as threatened [42]. Whether a better understanding of data deficient species and their true level of endangerment would substantially change the overall geographic patterns we see is unclear.

Insufficient locality data, and spatial biases in the data that are available, could affect the maps of biodiversity patterns. Neither the GBIF nor the *species*Link databases appear to be comprehensive for the locality data that exist. As well, they are conflicting in the spatial patterns of the data they do include. The differences likely reflect a differing set of museums and research databases integrated into the two systems, with neither yet being comprehensive. For the *species*Link database, a partial explanation may be that its origin was in São Paulo state through the Biota program, and so it was not originally a nationwide effort. Regardless though of the causes, given the large differences in the databases, what we can say about how sampling bias drives the perceived diversity patterns is limited. Others have found similar issues [43].

Accepting that the locality data are not ideal, this likely affects the species range maps. A range map is only as good as the data behind it, which are usually direct field observations. Because of insufficient exploration, some regions have few such observations and thus likely appear less diverse than they really are, particularly for the smaller-ranged or rare species. Conversely, range maps can also overestimate the presence of a species. Polygon ranges often do not capture the heterogeneity of suitable and unsuitable habitats within a species range. As well, there are certainly many cases where habitat loss has eliminated a species from its former range. With sufficient information and effort, it is possible to reduce such errors, and it is a recommended practice for finer-scale conservation planning [6,44,45]. Nevertheless, polygon range maps as we use here still tend to be the most comprehensive data available for broad-scale studies.

Improved integration of locality data into national and international databases would certainly aid the study of Brazilian biodiversity and help direct surveys to fill data gaps. A more comprehensive online platform is in development—the Brazilian Biodiversity Information System (SiBBr, www.sibbr.gov.br)—that will be the Brazilian node of GBIF. A hope is that this will result in a more comprehensive view of what data exist and where. Another ongoing initiative to integrate biodiversity data is the Program on Research of Biodiversity (Programa de Pesquisa em Biodiversidade—PPBio, ppbio.inpa.gov.br), which includes several regional nuclei representing different biomes. PPBio has three components: biodiversity surveys for long-term studies, support to develop biological collections, and thematic projects to develop methods for sustainable management of biodiversity.

Accepting that there are limitations in our knowledge, we do find that protected areas are in places with more species and importantly more of the most vulnerable species. This appears to be robust using various biodiversity metrics, although the correlations are weaker for small-ranged and endemic species and in general are weaker for fully protected versus sustainable use areas. It is possible that designation of a protected area itself will correlate with better documentation of biodiversity, because either the better knowledge of the area justified creation of the protected area or its creation resulted in a more through inventorying of the area. If either were the case, it might produce a pattern similar to what we observe. We have no practical way of testing for this effect or its magnitude. Nevertheless, it is encouraging that protected areas and biodiversity patterns correlate.

Much of the Brazilian biodiversity protection depends on sustainable use areas as, generally, they represent larger extents than the full protection areas. However, sustainable use areas often have not played an effective role in restricting threats to biodiversity, such as habitat loss and fragmentation, species invasion, and pollution [46]. In recent years, there has also been a worrying trend in Brazil with a pause in protecting new areas and even reductions in the extent and protection category of previously protected areas [47]. In the future, it will be important not only to allocate well any new protections according to biodiversity patterns, it is also essential to fully finance and empower protected areas to allow them to meet their goals.

Based on our findings, our recommendations are to:

1. Stop habitat loss and fragmentation of the Atlantic Forest, the center of much of the nation’s unique natural patrimony.
2. Implement and expand formal protection and restoration of the Atlantic Forest.
3. Set stricter rules and expand monitoring of biodiversity in sustainable use areas.
4. Transform sustainable use areas with high concentrations of endemic species into fully protected areas.
5. Focus resources for future biological studies on data deficient species and regions with inadequate sampling.
6. Support initiatives to collect and compile data on biodiversity (e.g., SiBBr and PPBio) using standard protocols as a way to fill gaps and to integrate knowledge (see Canhos et al. 2015)[48]. These initiatives should help and complement each other, as a way to optimize resources and to achieve conservations goals.
7. Continue to encourage and expand free and open access to information.

Biodiversity results, including GIS-ready datasets for open-access use, are available online at http://BiodiversityMapping.org and the Dryad Digital Repository: http://dx.doi.org/10.5061/dryad.6rv61.

## Supporting Information

S1 Fig Density of registered localities. Densities are per 1-degree grid cell from the Global Biodiversity Information Facility (GBIF) and from Brazil’s *species*Link site.
(TIF)

S1 File Abstract in Portuguese. (DOCX)

S1 Table Percent of each Brazilian state in protected areas or indigenous territories. FP—Full protection, SU—Sustainable Use, IL—Indigenous Land. Biome reflects only the predominant biomes found in the state: AF—Atlantic Forest, AM—Amazon, CA—Caatinga, CE—Cerrado, PP—Pampa, PT—Pantanal.
(DOCX)
