# Supplementary figures and images for: Patterns of Vertebrate Diversity and Protection in Brazil
Source: PLoS One. 2015 Dec 17;10(12):e0145064. doi: 10.1371/journal.pone.0145064 (PMC4682992; doi:10.1371/journal.pone.0145064)

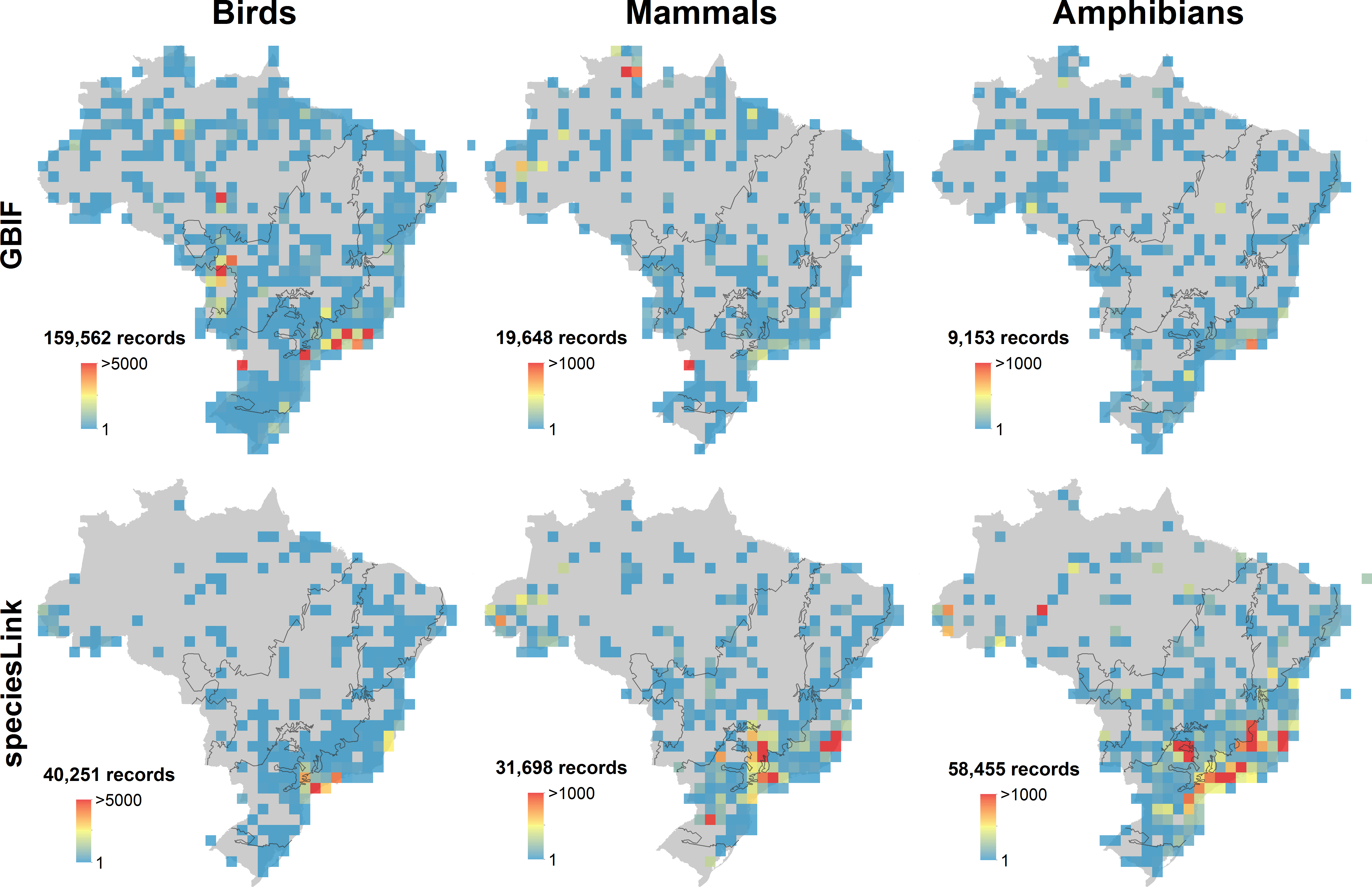

Supplement: S1 Fig — Densities are per 1-degree grid cell from the Global Biodiversity Information Facility (GBIF) and from Brazil’s speciesLink site. (TIF) [file pone.0145064.s001.tif]
