# Supplementary material for: Patterns of Vertebrate Diversity and Protection in Brazil
Source: PLoS One. 2015 Dec 17;10(12):e0145064. doi: 10.1371/journal.pone.0145064 (PMC4682992; doi:10.1371/journal.pone.0145064)
Supplement: S1 File — (DOCX) [file pone.0145064.s002.docx]

# **Resumo**

A maioria das decisões conservacionistas é tomada no nível nacional ou escalas espaciais mais finas. Prover informações úteis nessas escalas de tomada decisão é essencial para direcionar práticas de conservação. O Brasil é um dos países com maior megadiversidade e, consequentemente, decisões sobre conservação nesse país têm um impacto desproporcional na manutenção da diversidade global. Nós investigamos padrões geográficos de diversidade e proteção no Brasil para três grupos de vertebrados terrestres (aves, mamíferos e répteis), incluindo espécies endêmicas, de distribuição restrita e ameaçadas. Para entender as potenciais limitações dos dados, nós também exploramos como o viés espacial nas localidades de coleta podem influenciar os padrões de diversidade detectados. A riqueza mais elevada de espécies em geral se encontra na Amazônia e na Mata Atlântica, enquanto esta última domina em termos de espécies endêmicas do país e de distribuição restrita. Espécies ameaçadas globalmente não apresentam um padrão consistente. Padrões para aves foram similares aos de riqueza de espécies em geral, com maiores concentrações de espécies ameaçadas na Mata Atlântica, enquanto mamíferos mostraram um padrão mais difundido no país como um todo e uma elevada concentração na Amazônia. Poucos anfíbios constam na lista de espécies ameaçadas, ocorrendo principalmente na Mata Atlântica. Mamíferos com deficiência de dados ocorrem em todo o país, com concentração na Amazônia e sudeste da Mata Atlântica, não havendo aves categorizadas como deficientes de dados para o país. Por outro lado, aproximadamente um terço dos anfíbios é categorizado como deficiente de dados, com essas espécies espalhadas no país, mas havendo concentração detectada na porção mais sudeste. Entretanto, um viés espacial de dados de localidade das espécies possivelmente influencia os padrões de diversidade detectados. Regiões com pouca amostragem necessitam de mais estudos biológicos, assim como estes são necessários para as muitas espécies deficientes de dados. Todos os biomas, exceto a Amazônia, têm menos que 3% da sua área sob proteção integral. No entanto, as taxas de proteção se correlacionam com maior biodiversidade, incluindo elevados níveis de espécies ameaçadas e de distribuição restrita. Nossos resultados indicam a necessidade de ampliação de proteção formal no Brasil, especialmente na Mata Atlântica, com ênfase para áreas de proteção integral.
