# Supplementary material for: Patterns of Vertebrate Diversity and Protection in Brazil
Source: PLoS One. 2015 Dec 17;10(12):e0145064. doi: 10.1371/journal.pone.0145064 (PMC4682992; doi:10.1371/journal.pone.0145064)
Supplement: S1 Table — FP—Full protection, SU—Sustainable Use, IL—Indigenous Land. Biome reflects only the predominant biomes found in the state: AF—Atlantic Forest, AM—Amazon, CA—Caatinga, CE—Cerrado, PP—Pampa, PT—Pantanal. (DOCX) [file pone.0145064.s003.docx]

**S1 Table. Percent of each Brazilian state in protected areas or indigenous territories.** FP – Full protection, SU – Sustainable Use, IL – Indigenous Land. Biome reflects only the predominant biomes found in the state: AF – Atlantic Forest, AM – Amazon, CA – Caatinga, CE – Cerrado, PP – Pampa, PT – Pantanal.

| **State** | **code** | **Area (km^2^)** | **Biome** | **FP (%)** | **SU (%)** | **FP+SU (%)** | **IL (%)** | **FP+SU+IL (%)** |
| --- | --- | --- | --- | --- | --- | --- | --- | --- |
| Acre | AC | 164,166 | AM | 9.7 | 19.3 | 29.0 | 12.5 | 40.9 |
| Alagoas | AL | 27,769 | CA | 0.8 | 6.1 | 6.8 | 0.1 | 6.9 |
| Amazonas | AM | 1,559,105 | AM | 9.9 | 16.4 | 26.3 | 26.3 | 50.2 |
| Amapá | AP | 142,814 | AM | 33.5 | 29.2 | 62.7 | 8.3 | 70.8 |
| Bahia | BA | 564,720 | CE/AF | 1.6 | 8.2 | 9.8 | 0.2 | 9.9 |
| Ceará | CE | 148,913 | CA | 0.3 | 6.4 | 6.7 | 0.0 | 6.8 |
| Distrito Federal | DF | 5,801 | CE | 10.4 | 82.7 | 93.0 | 0.0 | 93.0 |
| Espírito Santo | ES | 46,071 | AF | 2.1 | 0.8 | 2.9 | 0.2 | 3.1 |
| Goiás | GO | 340,075 | CE | 0.9 | 4.4 | 5.3 | 0.0 | 5.3 |
| Maranhão | MA | 331,903 | AM/CA | 2.8 | 14.1 | 16.9 | 5.7 | 22.6 |
| Minas Gerais | MG | 586,531 | CE | 1.9 | 3.9 | 5.8 | 0.1 | 5.9 |
| Mato Grosso do Sul | MS | 357,127 | PT/CE | 0.8 | 2.0 | 2.8 | 1.8 | 4.6 |
| Mato Grosso | MT | 903,370 | CE/AM | 3.3 | 1.3 | 4.6 | 13.3 | 17.7 |
| Pará | PA | 1,247,823 | AM | 10.2 | 22.0 | 32.3 | 21.5 | 53.7 |
| Paraíba | PB | 56,450 | CA | 0.1 | 0.3 | 0.3 | 0.5 | 0.8 |
| Pernambuco | PE | 98,141 | CA | 0.7 | 3.5 | 4.2 | 1.2 | 5.4 |
| Piauí | PI | 251,582 | CA | 5.3 | 5.9 | 11.1 | 0.0 | 11.1 |
| Paraná | PR | 199,322 | AF | 2.2 | 6.2 | 8.4 | 0.3 | 8.7 |
| Rio de Janeiro | RJ | 43,715 | AF | 6.4 | 11.4 | 17.9 | 0.1 | 17.9 |
| Rio Grande do Norte | RN | 52,808 | CA | 0.2 | 1.3 | 1.5 | 0.0 | 1.5 |
| Rondônia | RO | 237,554 | AM | 14.0 | 9.1 | 23.1 | 20.5 | 38.8 |
| Roraima | RR | 224,292 | AM | 5.3 | 15.7 | 20.9 | 46.1 | 66.5 |
| Rio Grande do Sul | RS | 268,799 | PP | 0.4 | 1.9 | 2.3 | 0.2 | 2.6 |
| Santa Catarina | SC | 95,719 | AF/PP | 2.6 | 1.2 | 3.7 | 0.4 | 4.1 |
| Sergipe | SE | 21,907 | CA | 0.9 | 0.0 | 0.9 | 0.2 | 1.1 |
| São Paulo | SP | 248,209 | AF/CE | 3.9 | 10.7 | 14.6 | 0.1 | 14.7 |
| Tocantins | TO | 277,628 | CE/AM | 5.7 | 7.6 | 13.3 | 8.5 | 20.5 |
| Brazil |  | 8,502,314 |  | 6.0 | 10.8 | 16.8 | 12.2 | 28.3 |
